# Successful treatment of recurrent refractory Kaposi’s sarcoma in AIDS: a case report

**DOI:** 10.3389/fonc.2024.1462381

**Published:** 2024-12-20

**Authors:** Feng Li, Rongrong Zheng, Jun Yan, Zhongdong Zhang, Hong Liu, Jinchuan Shi

**Affiliations:** ^1^ The Second Infectious Disease Department, Xixi Hospital of Hangzhou, Hangzhou, China; ^2^ Department of pathology, Xixi Hospital of Hangzhou, Hangzhou, China

**Keywords:** Keywords: AIDS-related Kaposi’s sarcoma, PD-1 inhibitor, immunotherapy, complete remission, case report

## Abstract

Kaposi’s sarcoma (KS) is a soft tissue lesion that resembles a hyperpigmented angiosarcoma and is typically associated with human herpesvirus 8 (HHV-8) infection. It is most frequently observed in immunocompromised patients, particularly those with AIDS, and is also referred to as HIV-associated Kaposi’s sarcoma (AIDS-KS). The disease progresses rapidly, is challenging to manage, and has a high mortality rate. This case report presents a patient with AIDS-KS who experienced relapse after chemotherapy with anthracyclines. Subsequent chemotherapy with the same method had no significant effect. However, complete remission was achieved after the addition of a programmed cell death protein 1(PD-1) inhibitor, as confirmed by pathological biopsy. The PD-1 inhibitor was well-tolerated and had few adverse effects. It also helped to improve the immune reconstitution of the patient. The report highlights the remarkable efficacy of the PD-1 inhibitor in treating AIDS-KS. This provides case support for PD-1 inhibitors for AIDS-KS.

## Introduction

Kaposi’s sarcoma (KS) is a pigmented, angiosarcoma-like soft-tissue lesion that is commonly associated with human herpes virus 8 (HHV-8) infection. KS typically affects the skin, mucous membranes, lymphatic system, and gastrointestinal tract, and is most frequently observed in patients with compromised immune systems. The four main subtypes of KS are classical, African, transplantation, and AIDS-related.

KS is a common malignancy in patients with AIDS and is classified as an AIDS-related malignancy (1), also known as AIDS-KS, commonly arises in the advanced stages of HIV infection in affected individuals (2). The incidence of KS is 20,000 times higher among HIV patients who do not receive highly active antiretroviral therapy (HAART) (3). AIDS-KS typically affects the upper body and can cause damage to internal organs in addition to the skin. The disease progresses rapidly, is difficult to treat, and has a high mortality rate.

In recent years, there has been an increasing amount of research on the use of PD-1 inhibitors for treating various malignant tumors. However, there is limited data available on their effectiveness in treating KS. A case report describes an 82-year-old man with metastatic KS who achieved complete remission after 12 sessions of treatment with navulizumab, despite not being HIV-positive (4). In a phase II clinical trial studying the PD-1 inhibitor pembrolizumab for treating classic KS, 2 patients achieved complete remission, 10 had partial remissions, and 5 had stable disease after 204 months of follow-up. These results demonstrate that the PD-1 inhibitor has better therapeutic efficacy in patients with KS (5).but the efficacy of PD-1 inhibitors in the treatment of the AIDS-KS population is not clear. Here, we present a patient with AIDS-KS who achieved complete remission with the application of tirilizumab.

## Case presentation

**Figure 1:**
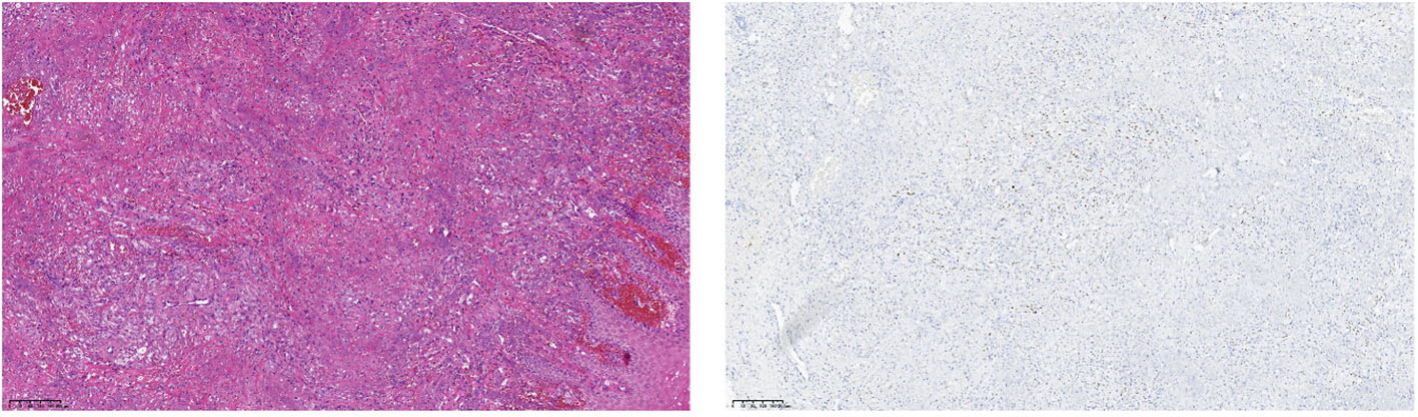
(Right plantar swelling)KS with fungal infection. Immunohistochemistry: CD34 (vascular +), CD58 (histiocytosis +), D2-40 (neovascular +), HHV8 (+), Ki-67 (partially +), PAS (+), antacid (-), hexaammonia silver staining (-).

A male patient, aged 30, presented with a facial rash that had been gradually increasing. He had been diagnosed with eczema and infectious dermatitis in several dermatology departments and had been treated with fusidic acid cream and loratadine tablets, but with no improvement. The rash then worsened, spreading to the neck and upper limbs. The patient presented with fever, tightness in the chest, and shortness of breath after activity. He was admitted to our hospital on 31st December 2022 and diagnosed with AIDS. Blood cultures were positive for Talaromyces marneffei, and cervical lymph node aspiration biopsy was consistent with lymph node tuberculosis. The blood test for tolulized red unheated serum test (TRUST)was 1:16. During hospitalization, a purplish-red mass measuring approximately 2cm x 3cm with a hard texture grew on the right sole of the foot. A surgical-assisted mass excision was performed, and biopsy pathology confirmed it to be consistent with KS (**Figure 1**).

The patient’s CD4+ T-lymphocyte count was initially only 3 cells/μL, and their HIV-RNA count was 3.26×10^5^copies/ml. They received highly effective antiretroviral therapy(HARRT)with tenofovir + lamivudine + dolutegravir, amphotericin B for t Talaromyces marneffei infections, isoniazid, moxifloxacin, and rifabutin for anti-lymphatic node tuberculosis, and benzylpenicillin for syphilis. After two months of hospitalization, the patient’s CD4+ T-lymphocyte count increased to 23 cells/μL, HIV-RNA was undetectable, the generalized rash subsided and disappeared, and the uncomfortable symptoms such as fever, chest tightness, and shortness of breath improved significantly, enabling the patient to carry out daily activities. For the treatment of KS, doxorubicin liposome was administered at a dose of 40mg every three weeks. After six consecutive applications, the purplish-red mass on the sole of the foot disappeared. The treatment course ended on 26th April 2023 and the patient achieved a significant improvement.

After one month of follow-up, the patient did not experience a recurrence of the generalized rash and fever. However, on 26th May 2023, the patient returned to the clinic with a purplish-red macular rash on the left thigh root and both calves. The rash was accompanied by a hard nodule on the thigh root and thickening of the leg circumference. Additionally, the patient experienced obvious swelling in both calves after activity. A skin biopsy was performed on the thigh maculopapular rash, and the biopsy’s pathological evaluation was consistent with KS, the immunohistochemical results were as follows: CD34 (-), D2-40 (+), ERG (+), HHV8 (+), Ki-67 (80%+), S-100 (-), Vimentin (+). The diagnosis was a recurrence of KS, and doxorubicin liposome 40 mg/dose was administered again, once every three weeks. After five consecutive applications, there were no obvious changes in the patient’s lower limb maculopapular rash. At present, the CD4+ T-lymphocyte count was 51 cells/μL and HIV-RNA was below the lower limit of detection.

**Figure 2:**
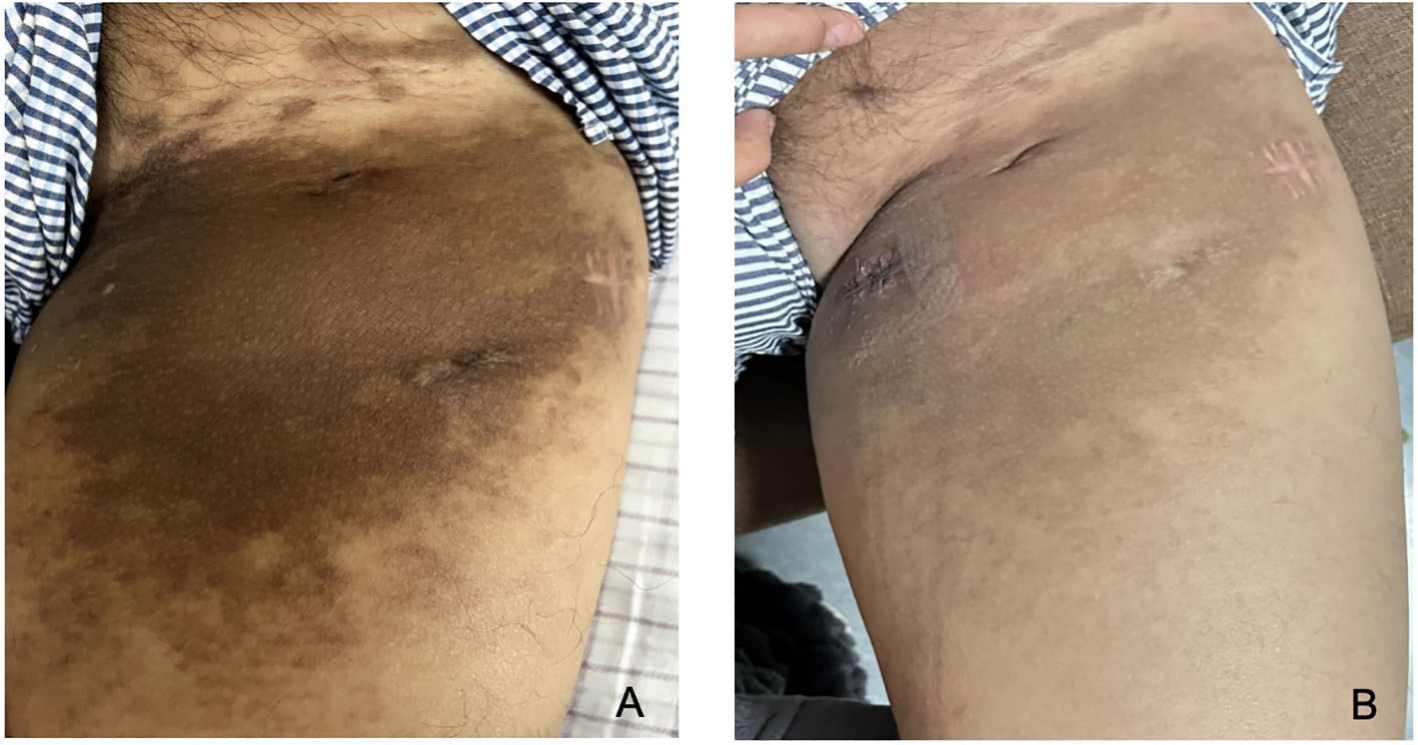
Before the addition of tirilizumab **(A)**, End of treatment **(B)**.

**Figure 3:**
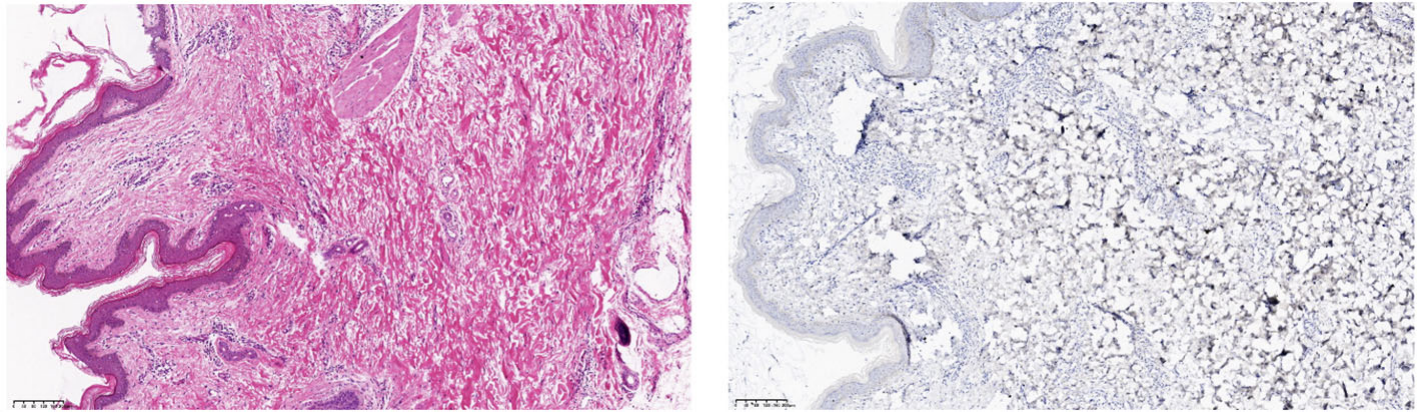
(Skin of left calf and thigh) Chronic vasculitis. Immunohistochemistry: CD34 (vascular +), D2-40 (vascular +), ERG (vascular endothelial +), HHV-8 (-), Ki-67 (few +).

**Figure 4:**
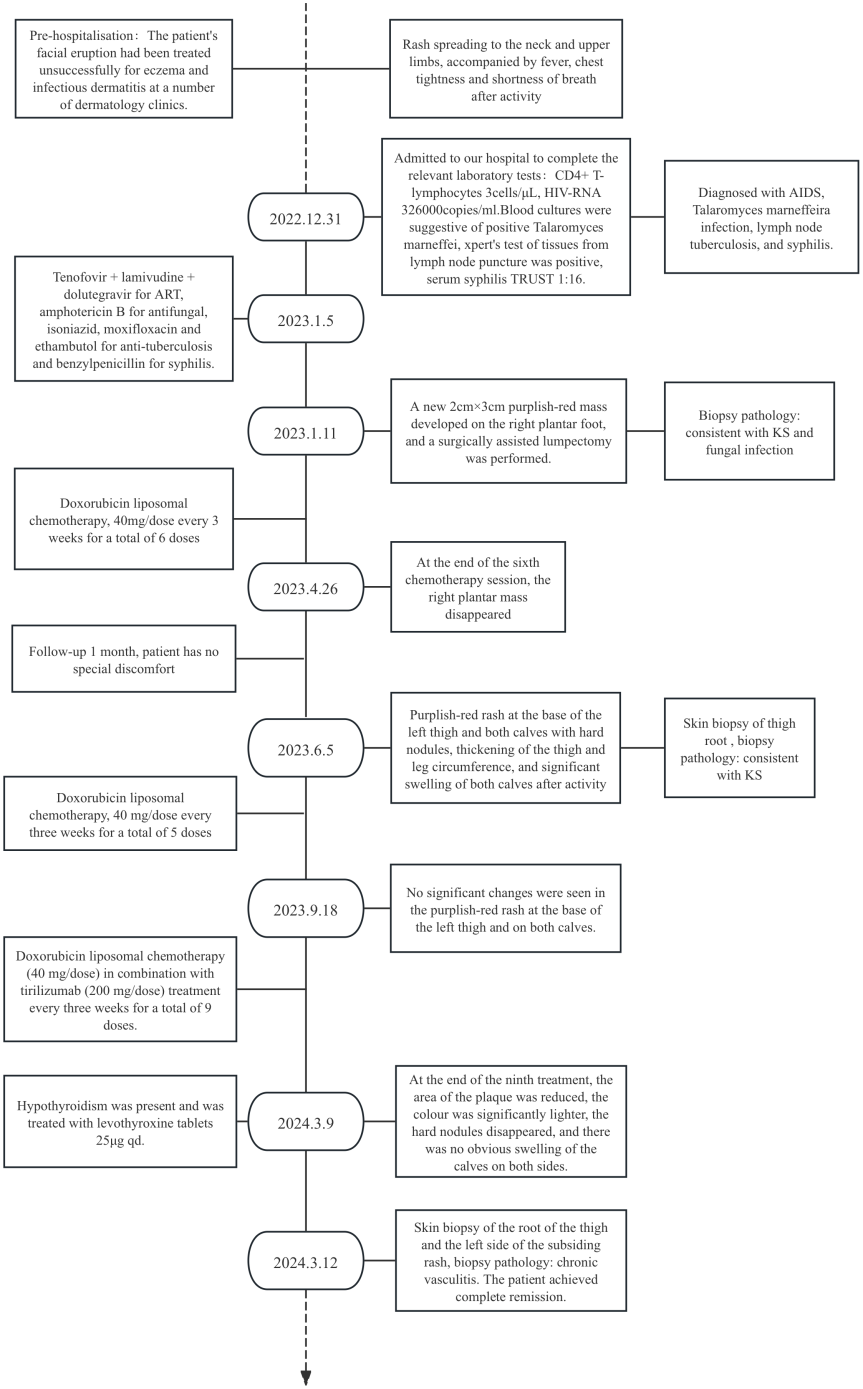
The timeline shows the entire diagnosis and treatment process of this case.

After obtaining informed consent from the patient, tirilizumab 200 mg/dose was added to the original regimen on September 19, 2023. The medication was administered once every three weeks. After nine consecutive uses, the patchy rash at the root of the left thigh reduced in range, the hard nodules disappeared, and the color became significantly lighter (**Figure 2**). Additionally, there was no swelling in both calves, indicating a promising therapeutic effect. Despite the absence of symptoms indicative of thyroid dysfunction, namely weakness, fatigue, facial swelling, or muscle pain, thyroid function tests revealed an elevated thyrotropin (TSH) level of 16.19 μIU/mL, a triiodothyronine (T3) level of 1.51 nmol/L, and a total thyroxine (T4) level of 107.09 nmol/L. The endocrinologists diagnosed PD-1-induced hypothyroidism and prescribed 25 μg levothyroxine tablets for treatment. To assess the therapeutic effect, skin biopsies were performed on the macules on the root of the thighs and on the calf of the left lower limb, respectively. Pathological assessment revealed chronic vasculitis (**Figure 3**). and the patient achieved complete remission. The timeline of patient diagnosis and treatment is shown in **Figure 4**.

## Discussion

The optimal treatment of AIDS-KS is yet to be standardized. In cases of only local skin lesions, alitretinoin, vincristine and bleomycin are all used for local treatment, and skin lesions are highly sensitive to radiotherapy, with complete remissions observed in about 95% of cases (6). However, local treatment is rarely used because it has no effect on systemic disease and radiation therapy may cause long-term local toxicity. Timely and effective highly active antiretroviral therapy (HAART) can significantly reduce the incidence of AIDS-KS (7), but some patients are unable to avoid AIDS-KS despite HIV viral load suppression and impressive T-lymphocyte counts (8), and HIV may be directly involved in carcinogenesis early in the human body. HAART combined with systemic chemotherapy is the current preferred treatment approach for AIDS-KS, with liposomal anthracyclines being the favored option. The combined use of HAART and liposomal anthracycline chemotherapy in advanced KS achieves a 70% overall remission rate and prolonged remission. However, it is unclear whether the treatment provides long-term benefits, and prolonged regimens increase the risk of adverse events, which limits the treatment of relapsed patients (9).

In recent years, immunotherapy has become a popular research topic for various diseases. The recombinant programmed cell death protein 1/ligand L1 (PD⁃1/PD⁃L1) pathway is capable of hindering the effector phase of cancer-specific immune responses. Several studies have identified that PD-L1 expression intensifies during viral lysis and replication after Kaposi’s sarcoma-associated herpesvirus(KSHV) infects human mononuclear cells, indicating a novel immune evasion strategy for KSHV (10). The expression of HHV-8 viral antigen results in increased expression of PD-1 and PD-L1 on the surface of antigen-specific T cells. This, in turn, leads to higher response rates to anti-PD-1 and anti-PD-L1 drugs (11). It has been discovered that PD-1 expression in natural killer cells of KS patients is linked to functional natural killer cell exhaustion. This, in turn, promotes the immune escape of tumor cells (12). Anti-PD-1 or anti-PD-L1 antibodies can block the interaction between PD-1 and PD-L1, which lifts the inhibition of T-lymphocyte proliferation.

**Figure 5:**
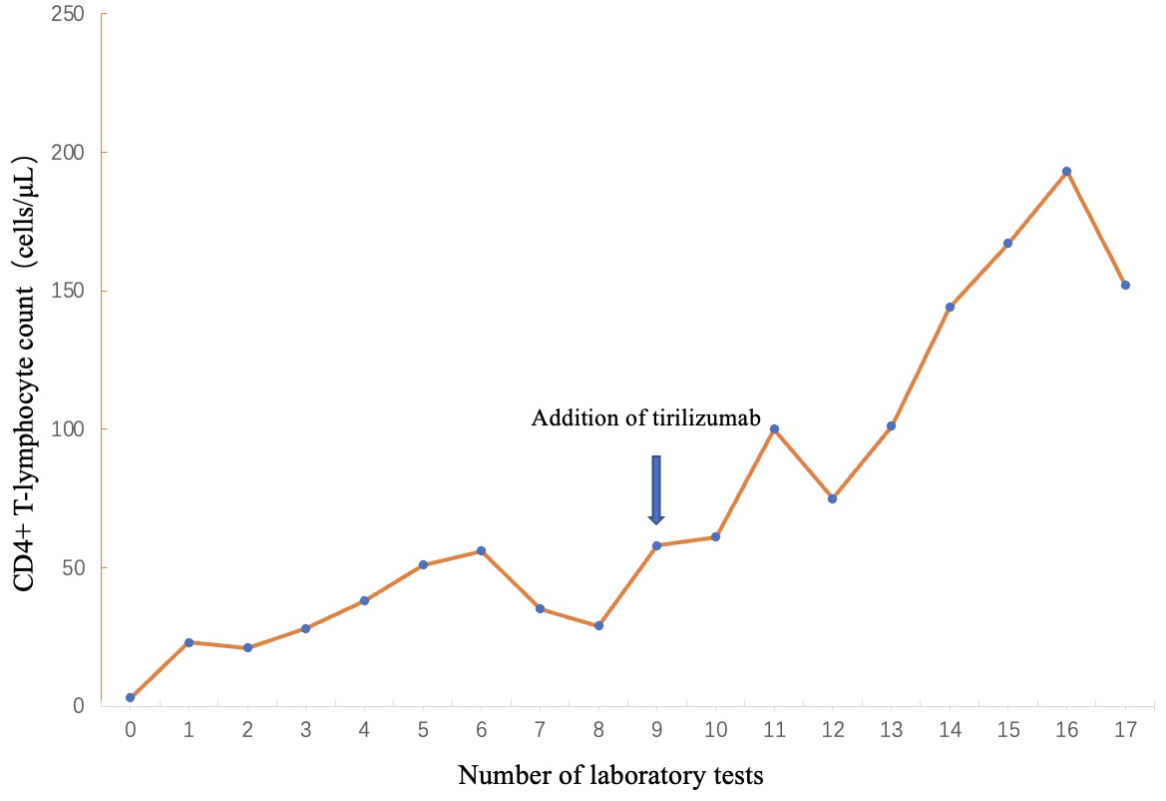
Trends in CD4+T-lymphocyte counts.

This reversal of the tumor immune microenvironment reactivates the ability of T-lymphocyte to lyse cancer cells. PD-1 inhibitors promote the proliferation of CD4+ T lymphocytes, which is consistent with the clinically observed changes in CD4+ T lymphocytes (**Figure 5**). It is speculated that improved HIV immune reconstitution promotes immune-mediated enhanced control and clearance of HHV-8, providing another possible avenue for AIDS-KS treatment.

The use of PD-1/PD-L1 inhibitors is theoretically more appropriate for patients with HIV-associated cancers, but in practice the unknown adverse effects that may be caused by the immunocompromised state of HIV patients have limited their use in this population. In this case, the patient had multiple opportunistic infections and had been taking multiple medications orally for an extended period. repeated application of PD-1 inhibitors and chemotherapy resulted in a gradual increase in the number of CD4+ T-lymphocyte, and a complete remission was achieved for relapsed and refractory AIDS-KS. Only mild malaise and fatigue were observed throughout the course of treatment, along with elevated thyroid-stimulating hormone. No other grade 2 or higher immune-related adverse reactions occurred. In addition to good efficacy, the encouraging results were attributed to fewer side effects and better tolerability.

In patients with AIDS-KS, vigilance is required for Kaposi Inflammatory Cytokine Syndrome (KICS), a serious disease state associated with KS that has only been proposed in recent years. KICS is characterized by a systemic inflammatory response and excessive release of multiple inflammatory cytokines. Clinical manifestations include skin lesions such as purplish-red or dark blue plaques, nodules or tumors, which may Systemic symptoms may also manifest, including fever, fatigue, and weight loss. Additionally, lymph node enlargement and organ dysfunction may occur. KICS is prone to occur in AIDS patients with a lower CD4+ cell counts, often accompanied by HHV-8 infections. The inflammatory milieu of KICS may facilitate the progression of Kaposi’s sarcoma (13). Additionally, both KICS and immune reconstitution syndrome (IRIS) are associated with human immunodeficiency virus (HIV) infection and an abnormal immune system response. These two conditions may co-occur in HIV-infected individuals and may also influence each other. The recovery of the immune system may intensify the reactivity of the inflammatory response, resulting in an exacerbation of KICS symptoms. Conversely, the inflammatory response associated with KICS may influence the development of IRIS, thereby complicating the recovery of the immune system. With a clinical mortality rate of up to 60% (14), KICS requires more accurate identification by clinicians to prevent misdiagnosis and to enhance clinical outcomes.

## Conclusion

Due to the complex pathogenesis of AIDS-KS, outdated basic research and the lack of specific therapeutic approaches, the treatment of AIDS-KS continues to be a challenge. The PD-1 inhibitor may enhance the proliferation of CD4+ T lymphocytes and improve immune reconstitution. This could potentially serve as a novel approach for successful treatment. This treatment shows promise due to its efficacy, few adverse effects and good tolerability. However, this study is only a case report of a clinical treatment, which has significant limitations and requires further exploration and research.

## Data Availability

The original contributions presented in the study are included in the article/supplementary material. Further inquiries can be directed to the corresponding author.
